# Using gene therapy to circumvent limitations of TRAIL-based cancer therapy

**DOI:** 10.1016/j.omton.2024.200844

**Published:** 2024-07-13

**Authors:** Ling Yin

**Affiliations:** 1 College of Medicine, University of Florida, Gainesville, FL, USA

## Main text

Tumor necrosis factor (TNF)-related apoptosis-inducing ligand (TRAIL), also known as Apo-2 ligand (Apo2L), is a 20-kDa type II transmembrane protein that belongs to the TNF superfamily. TRAIL activates apoptotic cell death through binding to DR5 receptors and caspase signaling cascade 3/7. As a death ligand, TRAIL induces apoptosis in transformed cells but shows low or no toxicity in normal cells.^1^ In fact, TRAIL expressed on immune cells plays important roles immunosurveillance of tumor cells.^2^ For these reasons, the specific cancer-targeting capability of TRAIL has attracted major interest in oncology as a potential candidate for cancer therapy.

Unfortunately, there are several barriers to the use of TRAIL-based cancer therapy.^3^^,^^4^^,^^5^ TRAIL itself has a very short half-life, which limits its therapeutic potential and pharmaceutical application. Recombinant TRAIL-based therapeutics exhibit low apoptotic potency, which is due to the inability of TRAIL to form native homotrimer complexes. TRAIL-based therapeutics exhibit low stability and accelerated aggregation at high concentrations, which limit drug dosages and cause adverse effects in clinical studies. Furthermore, TRAIL resistance can occur due to low expression of DR4/5, downregulation of pro-apoptotic proteins, and upregulation of apoptosis suppressors.

In a recent article published in *Molecular Therapy Oncology*, “Targeted treatment of chondrosarcoma with a bacteriophage-based particle delivering a secreted tumor necrosis factor-related apoptosis-inducing ligand (TRAIL),” the authors utilized a novel bacteriophage-derived particle (RGD4C.PDP-*sTRAIL*) to selectively deliver to tumor cells the transgene encoding a secreted TRAIL, effectively causing suicide and fratricide of surrounding tumor cells. They targeted tumors by displaying on the surface of bacteriophage (phage)-derived particles (PDPs) five copies of the tumor-targeting double cyclic CDCRGDCFC (RGD4C) ligand, which bind specifically to αvβ3 or αvβ5 integrin receptors on cancer cells and neoendothelial cells. RGD4C.PDP also displays the endosomal escape peptide to enhance gene delivery. *In vitro*, they detected high expression of TRAIL receptor 2/DR5 on human chondrosarcoma cells (99.8%) but not normal human articular chondrocytes (2.32%), making chondrosarcoma a particularly suitable target for TRAIL-based therapy. They demonstrated that RGD4C.PDP-*sTRAIL* selectively induced transgene expression in human chondrosarcoma tumors in mice and caused tumor regressions without any concerning safety signals.

The results of this study indicate targeted expression of secreted TRAIL may be a promising treatment for chondrosarcoma and other TRAIL-sensitive cancers, The wider implication of the study is that TRAIL expression via gene therapy can overcome many of the limitations of recombinant TRAIL therapeutics by avoiding dose and solubility issues and achieving high local intratumoral concentrations.
